# Urothelial Carcinoma Recurrence in an Orthotopic Neobladder without Urethral or Upper Urinary Tract Involvement

**DOI:** 10.1155/2019/8458706

**Published:** 2019-03-05

**Authors:** Chirag P. Doshi, Guliz A. Barkan, Marcus L. Quek

**Affiliations:** ^1^Department of Urology, Loyola University Medical Center, Maywood, IL, USA; ^2^Department of Pathology, Loyola University Medical Center, Maywood, IL, USA

## Abstract

We describe a case of a 71-year-old male with an isolated recurrence of urothelial carcinoma in an ileal neobladder without involvement of the upper urinary tract or urethra. He was diagnosed with high grade urothelial carcinoma involving a bladder diverticulum with associated carcinoma in situ. He underwent a radical cystectomy and orthotopic Studer ileal neobladder. On routine follow-up, 11 years following cystectomy, voided urine cytology was positive for high grade urothelial carcinoma. Further workup revealed normal upper urinary tracts, normal urethra, and a solitary lesion at the left anteroinferior wall of the neobladder. He subsequently underwent resection of the neobladder and conversion to an ileal conduit with pathology confirming the diagnosis of high grade urothelial carcinoma. Isolated recurrence of urothelial carcinoma within a neobladder without involvement of the upper urinary tract or urethra is rare. No guidelines exist regarding its management. Herein we present our management as well as the current literature published on this topic.

## 1. Introduction

The orthotopic ileal neobladder has become the preferred method of continent urinary diversion following radical cystectomy for bladder cancer. Urothelial carcinoma recurrence has been widely reported involving the urethra [1–3], upper urinary tract [4], or the ureteroenteric anastomosis [5–7]. However, isolated recurrences within the neobladder without concomitant disease in the remnant urothelium are exceedingly rare, with only 3 other case reports in the literature (Table 1). Here we present a patient with an isolated urothelial carcinoma recurrence in the neobladder 11 years after radical cystectomy.

## 2. Case Report

A 71-year-old gentleman who was diagnosed at age of 59 with high grade urothelial carcinoma involving a bladder diverticulum with associated carcinoma in situ underwent a radical cystectomy, prostatectomy, extended lymphadenectomy, and orthotopic Studer ileal neobladder. Pathology confirmed a high grade urothelial carcinoma arising in a right-sided diverticulum with extension into the perivesical fat and associated carcinoma in situ. There was no evidence of extension into the urethra or ureters and all surgical margins were negative. None of the 40 lymph nodes removed showed carcinoma (pathologic stage pT3aN0Mx). He underwent adjuvant chemotherapy (methotrexate, vinblastine, doxorubicin, and cisplatin), but only tolerated 2 cycles before discontinuing due to side effects. He continued to have surveillance imaging and follow-up every 6-12 months.

**Figure 1:**
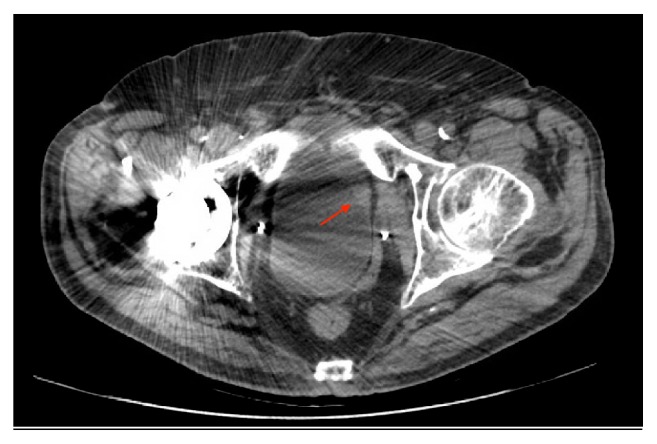
CT imaging with focal mural thickening involving the left anteroinferior neobladder.

On routine follow-up, 11 years following cystectomy, voided urine cytology was positive for high grade urothelial carcinoma. Follow-up CT imaging showed normal upper tracts and interval development of focal mural thickening involving the left anteroinferior neobladder (Figure 1). He underwent a cystoscopy, which revealed a normal urethra, and a solitary lesion at the left anteroinferior wall of the neobladder. Biopsies of this mass revealed an invasive high grade urothelial carcinoma with focal squamous differentiation.

**Figure 2:**
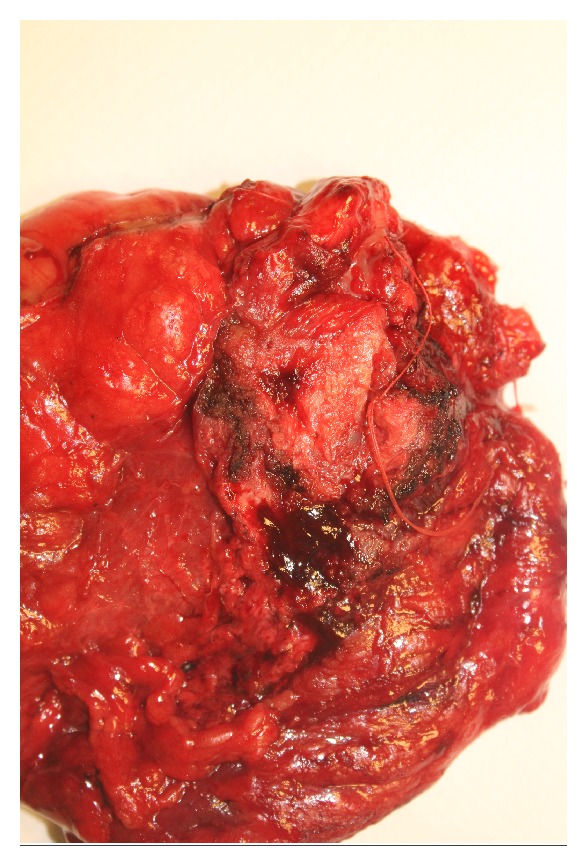
Gross pathology.

**Figure 3:**
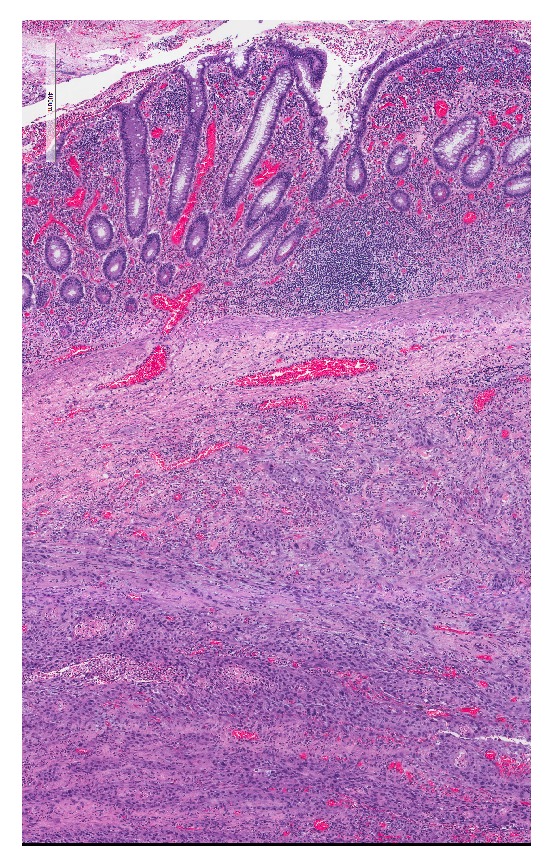
Microscopic pathology.

He subsequently underwent resection of the orthotopic neobladder and conversion to an ileal conduit. Intraoperatively, the mass involved the neobladder with possible extension into the pelvic sidewall and pubic bone periosteum. All gross disease was resected. Frozen section margins from pubic bone periosteum and urethra were negative. On gross examination of the final pathologic specimen, a 4.2cm x 2.3cm exophytic, friable mass was noted protruding into the neobladder lumen. Upon sectioning, this mass was noted to have variable depths of invasion, the highest being 9mm through the mucosa, submucosa, and serosa with involvement of the surrounding adipose tissue. Final pathology confirmed high grade urothelial carcinoma involving the neobladder and extending into the surrounding fat (Figures 2 and 3). No tumor was seen at the neobladder-urethral anastomosis and all surgical margins were negative.

## 3. Discussion

An isolated recurrence of urothelial carcinoma in an ileal neobladder without involvement of the upper tract or urethra is rare. To our knowledge, only 3 case reports have been previously published in the literature (Table 1) [9–11]. Patients may present with new hydronephrosis and gross hematuria or as in our case have an abnormal urine cytology. Management of the previously reported cases included transurethral resection in two and partial neobladder resections in the other.

The mechanism of local recurrence in the mucosa of the ileal neobladder is unclear. The development of recurrent solitary or multifocal tumors in the urinary tract is a well-known characteristic of urothelial cell carcinoma. After urinary diversion, recurrences have been reported to occur in the urethra [1–3], upper urinary tract [4], and ileal conduit [6], at the ureteroenteric anastomosis [5–7], as well as colonic mucosa of an ileocolonic neobladder [12]. Direct invasion and implantation are the two proposed hypotheses for urothelial carcinoma recurrence in any part of the urinary tract, including segments of bowel used for urinary diversion. Although some studies have provided evidence for the polyclonal origin of urothelial carcinoma, supporting the idea that multiple tumor recurrences might be the result of carcinogens in the urine, most molecular studies have favored the monoclonal origin, implicating an intraepithelial spread or intraluminal seeding as the source of multiple synchronous or metachronous urothelial tumors [12].

## 4. Conclusion

We report a rare case of urothelial carcinoma recurrence without involvement of upper tracts, ureteroileal anastomosis, or urethra, in an orthotopic ileal neobladder 11 years after radical cystectomy. Our patient underwent resection of the neobladder and conversion to an ileal conduit. At his most recent follow-up visit 3 months later, he continued to be without evidence of recurrence or metastasis. No guidelines exist regarding the management of urothelial carcinoma recurrence. Tumor recurrences have been reported up to 12 years from initial cystectomy, making it imperative that these patients have long-term follow-up.

## Figures and Tables

**Table 1:** Cases of urothelial cell carcinoma recurrence in ileal neobladder.

Paper	Patient age/sex	Recurrence site	Urethral recurrence	Upper tract recurrence	Treatment for recurrence
Kawamoto et al. [5]	61y/M	Uretero-ileal anastomosis	No	Yes	Left nephroureterectomy, resection of neobladder, urethrectomy

Ide et al. [6]	73y/M	Uretero-ileal anastomosis, urethral-neobladder anastomosis	Yes	Yes	Left nephroureterectomy, resection of neobladder, urethrectomy

Hadzi-Djokic et al. [7]	65y/M	Uretero-ileal anastomosis	No	Yes	Right nephroureterectomy, partial resection of neobladder

Moore et al. [8]	62y/M	Neobladder (multiple sites), urethral-neobladder anastomosis, urethra	Yes	No	Resection of neobladder, urethrectomy, creation of ileal conduit

*Yamashita et al. [9] *	*66y/M*	*Neobladder (multiple sites)*	*No*	*No*	*Transurethral resection + intravesical BCG*

*Cakmak et al. [10]*	*40y/M*	*Neobladder (multiple sites)*	*No*	*No*	*Transurethral resection*

*Cherbanyk et al. [11]*	*66y/M*	*Neobladder*	*No*	*No*	*Partial resection of neobladder*

*Present case*	*71y/M*	*Neobladder-left anterior wall *	*No*	*No*	*Resection of neobladder, creation of ileal conduit*
